# Association between early life famine exposure and risk of metabolic syndrome in later life

**DOI:** 10.1111/1753-0407.13319

**Published:** 2022-09-29

**Authors:** Yi Zhang, Hongyan Qi, Chunyan Hu, Shuangyuan Wang, Yuanyue Zhu, Hong Lin, Lin Lin, Jie Zhang, Tiange Wang, Zhiyun Zhao, Mian Li, Yu Xu, Min Xu, Yufang Bi, Weiqing Wang, Yuhong Chen, Jieli Lu, Guang Ning

**Affiliations:** ^1^ Department of Endocrine and Metabolic Diseases, Shanghai Institute of Endocrine and Metabolic Diseases, Ruijin Hospital Shanghai Jiao Tong University School of Medicine Shanghai China; ^2^ Shanghai National Clinical Research Center for Metabolic Diseases, Key Laboratory for Endocrine and Metabolic Diseases of the National Health Commission of the PR China, Shanghai Key Laboratory for Endocrine Tumor, State Key Laboratory of Medical Genomics, Ruijin Hospital Shanghai Jiao Tong University School of Medicine Shanghai China

**Keywords:** famine, metabolic syndrome, obesity, 饥荒, 肥胖, 代谢综合征

## Abstract

**Background:**

Previous studies reported that famine exposure had an effect on metabolic syndrome (MetS). However, there is an inadequacy of study regarding the association between famine exposure, adulthood general obesity, and the risk of MetS.

**Methods:**

A total of 8883 subjects aged ≥40 years from Jiading community in Shanghai were included. We defined famine exposure subgroups as nonexposed (1963–1974), fetal exposed (1959–1962), childhood exposed (1949–1958), and adolescence exposed (1941–1948). MetS was defined based on the National Cholesterol Education Program Adult Treatment Panel III (NCEP‐ATP III) criteria.

**Results:**

Compared with the nonexposed group, the risks of MetS were increased in the fetal‐, childhood‐, and adolescence‐exposed groups with odds ratios (OR) and 95% confidence intervals (CI) of 1.48 (1.23–1.78), 1.89 (1.63–2.20), and 2.34 (1.99–2.74), respectively. After adjusting for sex, age, smoking status, drinking status, education, body mass index (BMI), and physical activity, the increased risk of MetS related to the fetal‐exposed and childhood‐exposed groups with OR and 95% CI of 1.42 (1.04–1.94) and 1.50 (1.02–2.21), respectively, were observed only in women. Famine exposure was significantly associated with MetS among individuals with a BMI < 23 kg/m^2^ (*p* for interaction between BMI categories and famine exposure = 0.0002 in the whole cohort), while there existed a gender difference (*p* = 0.0023 in females, *p* = 0.4484 in males). When evaluating the joint effects of the combination of famine exposure in early life and general obesity in adulthood on MetS, we observed the highest estimate in participants with both adulthood general obesity and fetal famine exposure (OR 17.52; 95% CI, 10.07–30.48) compared with those without famine exposure nor adulthood obesity.

**Conclusions:**

Obesity in adulthood significantly further aggravated the risk of MetS in individuals who experienced early life undernutrition, especially in females.

## Graphical abstract

**Highlights**

1. In a community‐dwelling Chinese population, famine exposure in early life, especially during the fetal and childhood stages, has an association with an increased risk of metabolic syndrome (MetS) in later life, but the above findings only applied to females, not males.
2. Compared to nonexposed and nonobese participants, those with both fetal famine exposure and adulthood general obesity had a higher risk of MetS of more than 17‐folds.
3. The coexistence of malnutrition in early life and adulthood overweight/obesity had an association with a higher risk of MetS.

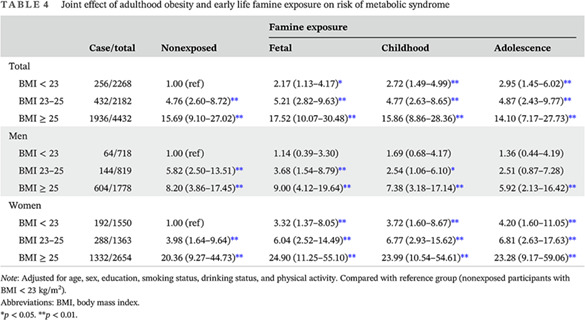

## 摘要

**背景:**

以前的研究报道了饥荒暴露对代谢综合征(MetS)有影响。然而, 关于饥荒暴露、成年期肥胖和 MetS 风险之间的关系则较少研究。

**方法:**

纳入上海市嘉定社区年龄 >40岁的8883名受试者。我们将饥荒暴露亚组定义为未暴露(1963‐1974)、胎儿暴露(1959‐1962)、儿童暴露(1949‐1958)和青春期暴露(1941‐1948)。 MetS根据 NCEP‐ATP III标准定义。

**结果:**

与未暴露组相比, 胎儿、儿童和青春期暴露组的 MetS 风险增加, OR 和 95% CI 分别为 1.48 (1.23‐1.78)、1.89 (1.632.20) 和 2.34 (1.99‐ 2.74)。在校正性别、年龄、吸烟状况、饮酒状况、教育程度、体重指数(BMI)和体力活动后, 女性中观察到胎儿暴露组和儿童暴露组发生 MetS 的风险增加, OR 和 95%CI 分别为 1.42( 1.04‐1.94) 和 1.50 (1.02‐2.21)。在 BMI<23 kg/m^2^ 的个体中, 饥荒暴露与 MetS 显着相关(整个队列中 BMI 类别与饥荒暴露之间的交互作用 P = 0.0002), 但存在性别差异(女性 P=0.0023, 男性P=0.4484)。在评估生命早期饥荒暴露和成年期肥胖对 MetS 的联合影响时, 我们观察到与没有饥荒暴露或成年肥胖的人相比, 成年肥胖和胎儿饥荒暴露的参与者有最高估计值(OR, 95%CI:17.52, 10.07‐ 30.48)。

**结论:**

成年期肥胖显著加剧了早年营养不良个体(尤其是女性)发生 MetS 的风险。

## 1 INTRODUCTION

Metabolic syndrome (MetS), known as a multiplex risk factor composed of multiple metabolism‐related factors, is characterized by dysglycemia, obesity, and other cardiovascular disease‐related risk factors.^1^ During the past few decades, a rapid increase of the prevalence of MetS has been noticed in China. A national study showed that the prevalence of MetS was 33.9% (36.8% in women and 31.0% in men). The above research suggested that there were approximately 454 million adults who had MetS in China.^2^ MetS increases the risk of diabetes, chronic kidney disease, and cardiovascular disease and is becoming an increasingly serious and common epidemic.

As living standards improve, the prevalence of child and adult obesity is also increasing. Obesity increases the population's susceptibility to metabolic diseases such as diabetes,^3^ hypertension,^4^ and MetS.^5^ In epidemiological studies, overweight and obesity identified by body mass index (BMI) associate consistently with the risk of MetS.^5^ The developmental origins of health and disease (DOHaD) theory posits that critical periods in early life (including fetal stage, childhood, and adolescence) environments, which affect growth, metabolism, and neurogenesis, are followed by later environments that determine the risk for metabolic disease.^6^, ^7^ Early malnutrition has a far‐reaching impact on the onset and development of metabolic disease in adulthood.^8^, ^9^, ^10^, ^11^ Compared with subjects who were not exposed to famine and were of normal weight, the prevalence of MetS was higher in populations with both famine exposure and adult overweight.^12^, ^13^, ^14^ Therefore, this suggests that there may be an interaction between famine exposure in the fetal and prenatal stages and adulthood obesity, thereby increasing the risk of MetS. However, significant interactions have not been found between early life famine exposure and adult BMI on the risk of MetS in the Dongfeng‐Tongji cohort^13^ and the China Health and Retirement Longitudinal Survey (CHARLS).^14^

The Great Chinese Famine during 1959 to 1961, which affected people living in the Chinese mainland, resulted in more than 30 million excess deaths.^15^ It led to a considerably long period of malnutrition for most people, and only in recent decades these people gradually completed the nutritional transition. Our former studies showed that famine exposure in early life may lead to a range of diseases, including nonalcoholic fatty liver disease,^10^ diabetes,^16^ and cardiovascular diseases.^9^ However, the effects of general obesity on the association between early life famine exposure and risk of MetS in later life remain unclear.

Using the data obtained from a Chinese community‐dwelling population,^17^, ^18^, ^19^ we aimed to evaluate the association between early life famine exposure and risk of MetS in later life and to further clarify the modifying effect of general obesity in adulthood on the association between early life famine exposure and risk of MetS.

## 2 RESEARCH DESIGN AND METHODS

### 2.1 Study population

Subjects in the present research were derived from a community‐dwelling cohort in Jiading District, Shanghai, China.^20^, ^21^ During February to August 2010, 10 569 residents aged ≥40 years were recruited by door‐to‐door visits or telephone calls. A total of 10 375 residents participated in the study. Participants who were born before 1 January 1941 were excluded (adulthood exposed; *n* = 1492), leaving 8883 subjects in the current analysis. A flow chart of this study is presented in Figure S1. The research protocol was approved by the Human Research Ethic Committee of Ruijin Hospital, Shanghai Jiao Tong University School of Medicine. All subjects provided written informed consent in advance.

### 2.2 Measurements

Standardized questionnaires were used to document medical history, sociodemographic data, and lifestyle (drinking status, smoking status, and physical activity). When measuring height and weight, participants were asked to wear light clothes and take off their shoes, their chests and abdomen facing straight forward, eyes looking straight ahead, heels, sacrum and shoulder blades standing close to the height meter post. Weight and height were measured to the nearest 0.1 kg and 0.1 cm, respectively. Waist circumference (WC) was measured by the horizontal circle's circumference centered on the navel when the subjects were standing upright, and WC measured to the nearest 0.1 cm. The calculation method of the BMI (kg/m^2^) was body weight (kg) divided by height (m) squared. The measurement of blood pressure was conducted by trained professionals. Exercise, smoking, drinking coffee, tea, etc. were avoided in the first half hour of measurement, and the measurement was started after 5 to 10 minutes of quiet rest. During the measurement, the right upper limb was fully exposed, and the cuff was loose. An Omron automatic electronic sphygmomanometer was used for the measurement. Three repeated measurements were carried out, and the average value was taken.

According to the lifestyle risk factors in the questionnaire, the status of smoking was divided into never, past, and current smokers. Current smoking was defined as smoking at least one cigarette daily or seven cigarettes weekly in the past half a year. The status of drinking was categorized as never drinking, past drinking, and current drinking. Current drinking was defined as the regular behavior of consuming alcohol at least once weekly for the past half a year. According to the International Physical Activity Questionnaire‐Short Form,^22^ the intensity of physical activity was divided into below moderate, moderate, and vigorous physical activity. Moderate physical activity was defined as >150 minutes of moderate‐intensity physical activity per week and vigorous physical activity as >75 minutes of vigorous‐intensity aerobic physical activity per week or a combination of equal proportions of moderate‐ and vigorous‐intensity activity.

Before blood samples were collected, all subjects had to fast for at least 10 hours. The glucose oxidase method on an autoanalyzer was used to determine fasting plasma glucose (FPG). An autoanalyzer was used to measure triglycerides (TG) and high‐density lipoprotein cholesterol (HDL‐C).

### 2.3 Definitions

MetS was defined based on the National Cholesterol Education Program Adult Treatment Panel III (NCEP‐ATP III).^23^ If participants met three or more of the following criteria, we defined them as having MetS: (a) fasting TG levels of 1.7 mmol/L or above; (b) HDL‐C levels lower than 1.04 mmol/L in males and lower than 1.29 mmol/L in females; (c) FPG levels of 6.1 mmol/L or above or having been treated with antihyperglycemic medication or earlier diagnosis of diabetes; (d) WC of 102 cm or above in males and 88 cm or above in females; and (e) systolic blood pressure ≥130 mm Hg and diastolic blood pressure ≥85 mm Hg or having been treated with antihypertensive medication or a history of hypertension.

Overweight was defined with a BMI cutoff of 23 to 25 kg/m^2^ and obesity with a BMI cutoff of 25 kg/m^2^ or above as these cutoffs have been recommended as more suitable for Asian concepts of overweight and obesity.^24^, ^25^, ^26^

In the present study, based on the year of birth, subjects were divided into the four subgroups of fetal exposure (born in 1/1/1959–31/12/1962), childhood exposure (born in 1/1/1949–31/12/1958), adolescent exposure (born in 1/1/1941–31/12/1948), and nonexposed (born after 1/1/1963).^8^, ^9^, ^16^

### 2.4 Statistical analysis

All analyses were performed using SAS version 9.4 (SAS Institute Inc). Mean ± SD represent the continuous variables of normal distribution. Continuous variables of skew distribution are represented by medians (interquartile ranges). Categorical variables are expressed as total numbers (corresponding percentages). The odds ratios (OR) and 95% confidence intervals (CI) were calculated by a multivariable‐adjusted logistic regression model. The multivariable‐adjusted model included sex (for the whole population only), age (years), education (education level with junior high school and below or senior high school and above), smoking status (never, past, and current smoking), drinking status (never, past, and current drinking), and the intensity of physical activity (below‐moderate, moderate, and vigorous physical activity). Menstruation status and use of contraceptive drugs were further adjusted for when analyzing famine exposure and MetS in the female group. When analyzing the association between famine exposure and MetS across different BMI subgroups, BMI was further adjusted. We further assessed the association of the combination of BMI and famine exposure with the risk of MetS. The potential interaction between adulthood overweight/obesity and early life famine exposure in MetS was investigated by conducting multiplicative interaction analysis. A two‐tailed value of *p* < 0.05 was proved to be statistically significant.

## 3 RESULTS

### 3.1 Baseline characteristics

The baseline features of the 8883 individuals according to MetS status are displayed in Table 1. The prevalence of MetS was 29.54% (2624/8883) in the present study. The differences between participants with and without MetS were significant in all characteristics. Subjects with MetS were less likely to be current smokers, current drinkers, had higher levels of education, and were inclined to be physically active.

**TABLE 1:** Characteristics of 8883 participants with and without metabolic syndrome

	Metabolic syndrome (−)	Metabolic syndrome (+)	*p* value
No. of participants (%)	6259 (70.46)	2624 (29.54)	
Age, y	55.16 ± 7.39	57.14 ± 6.87	<0.0001
Male, *n* (%)	2504 (40.01)	812 (30.95)	<0.0001
Body mass index, kg/m^2^	24.38 ± 2.95	26.99 ± 3.16	<0.0001
Waist circumference, cm	80.44 ± 8.36	87.63 ± 8.08	<0.0001
Current smoker, *n* (%)	1455 (23.25)	454 (17.30)	<0.0001
Current drinker, *n* (%)	695 (11.46)	219 (8.62)	<0.0001
High school or above education, no. (%)	1534 (24.63)	495 (18.97)	<0.0001
Moderate and vigorous physical activity, no. (%)	875 (13.98)	414 (15.78)	0.0282
Triglycerides, mmol/L	1.17 (0.88–1.53)	2.15 (1.75–2.96)	<0.0001
High‐density lipoprotein cholesterol, mmol/L	1.4 ± 0.31	1.12 ± 0.23	<0.0001
Systolic blood pressure, mm Hg	135.4 ± 18.9	148.13 ± 17.34	<0.0001
Diastolic blood pressure, mm Hg	81.72 ± 10.06	86.79 ± 9.7	<0.0001
Fasting plasma glucose, mmol/L	5.22 ± 1.15	6.2 ± 1.85	<0.0001

*Note*: Data are mean ± SD or median (interquartile) for continuous variables or percentages for categorical variables.

### 3.2 Famine exposure in early life and MetS in later life

The association of famine exposure in early life with the risk of MetS in later life is presented in Table 2. In the multivariable‐adjusted logistic regression model, famine exposure in early life was positively associated with the risk of MetS in later life. Without adjusting for covariates, the OR (95% CI) of MetS were 1.48 (1.23–1.78), 1.89 (1.63–2.20), and 2.34 (1.99–2.74) for the total population with the fetal‐exposed, childhood‐exposed, and adolescent‐exposed groups, respectively. After adjustment for sex, age, education level, smoking status, drinking status, and the intensity of physical activity, famine exposure in the fetal stage (OR 1.23; 95% CI, 1.00–1.53) was associated with a higher risk of MetS in adulthood. However, with further adjustment for BMI, the increased risk of MetS was no longer noticeable within all famine exposure subgroups. Sensitivity analysis using modified Asian WC cutoffs (90 cm in men and 80 cm in women) of the NCEP ATP III^27^, ^28^ is shown in Table S1. The OR (95% CI) of MetS associated with fetal or childhood famine exposure compared to the nonexposed group were 1.34 (1.01–1.79) and 1.36 (0.94–1.97), respectively.

**TABLE 2:** Odds ratios (95% CI) for metabolic syndrome according to famine exposure in early life

	Nonexposed	Famine exposure		
		Fetal	Childhood	Adolescence
Whole cohort				
Case/total	265/1387	317/1225	1245/4029	797/2242
Model 1	1.00 (ref)	1.48 (1.23–1.78)**	1.89 (1.63–2.20)**	2.34 (1.99–2.74)**
Model 2	1.00 (ref)	1.19 (0.96–1.46)	1.12 (0.86–1.47)	0.99 (0.65–1.50)
Model 3	1.00 (ref)	1.23 (1.00–1.53)	1.13 (0.86–1.49)	1.02 (0.67–1.56)
Model 4	1.00 (ref)	1.18 (0.94–1.48)	1.13 (0.84–1.52)	1.03 (0.65–1.63)
Men				
Case/total	133/540	121/464	346/1413	212/899
Model 1	1.00 (ref)	1.08 (0.81–1.44)	0.99 (0.79–1.25)	0.94 (0.74–1.21)
Model 2	1.00 (ref)	0.99 (0.72–1.38)	0.81 (0.52–1.28)	0.67 (0.33–1.37)
Model 3	1.00 (ref)	0.99 (0.71–1.39)	0.81 (0.51–1.27)	0.66 (0.32–1.34)
Model 4	1.00 (ref)	0.98 (0.70–1.39)	0.82 (0.51–1.31)	0.68 (0.32–1.43)
Women				
Case/total	132/847	196/761	899/2616	585/1343
Model 1	1.00 (ref)	1.88 (1.47–2.41)**	2.84 (2.32–3.47)**	4.18 (3.37–5.18)**
Model 2	1.00 (ref)	1.41 (1.07–1.85)*	1.45 (1.02–2.05)*	1.33 (0.79–2.25)
Model 3	1.00 (ref)	1.51 (1.14–2.00)**	1.48 (1.04–2.11)*	1.41 (0.83–2.41)
Model 4	1.00 (ref)	1.42 (1.04–1.94)*	1.50 (1.02–2.21)*	1.44 (0.80–2.59)
Model 5	1.00 (ref)	1.41 (1.03–1.94)*	1.48 (0.97–2.24)	1.43 (0.78–2.63)

*Note*: Model 1, unadjusted. Model 2, adjusted for age and sex. Model 3, adjusted for age, sex, education, smoking and drinking status, and physical activity. Model 4, further adjusted for body mass index. Model 5, further adjusted for status of menstruation and use of contraceptive drugs.

* *p* < 0.05.

** *p* < 0.01.

The association between early life famine exposure and MetS risk differs between genders. With further adjustment for BMI, the fetal‐ (OR 1.42; 95% CI, 1.04–1.94) and childhood‐exposed (OR 1.50; 95% CI, 1.02–2.21) groups showed a higher risk of MetS in adult women, but not in men, compared with the nonexposed group. With further adjustments for menstruation status and contraceptive drug use in women, the fetal‐ (OR 1.41; 95% CI, 1.03–1.94) exposed group showed a higher risk of MetS compared with the nonexposed women. Similar results were observed when we carried out further analysis to examine the association of early life famine exposure and risk of MetS components. After adjustments for sex, age, education level, smoking status, drinking status, intensity of physical activity, and BMI, the fetal‐ and childhood‐exposed groups showed a higher risk of high fasting TG, and the fetal‐, childhood‐, and adolescence‐exposed groups showed a higher risk of high fasting glucose in adult women, but not in men, compared with the nonexposed group. (Table S2).

### 3.3 Early life famine exposure, adulthood obesity, and MetS

We did a stratified analysis by sex. Compared with the nonexposed group, the fetal‐exposed and childhood‐exposed groups had an association with a higher risk of MetS with OR (95% CI) of 2.32 (1.14–4.74) and 3.00 (1.27–7.10) in the lean group (defined as BMI < 23 kg/m^2^), after adjustment for covariates. Interactions between famine and BMI were found to increase the risk of MetS (*p* = 0.0002). Additionally, a significant interaction on the risk of MetS between famine exposure subgroups and BMI was only observed in women but not in men (*p* value for interaction was 0.0023 in women, 0.4484 in men) (Table 3),

**TABLE 3:** Associations between early life famine exposure and adulthood obesity with the risk of metabolic syndrome

	Case/total	Nonexposed	Famine exposure			*p* for interaction
			Fetal	Childhood	Adolescence	
Total						
BMI < 23	256/2268	1.00 (ref)	2.32 (1.14–4.74)*	3.00 (1.27–7.10)*	3.51 (0.98–12.57)	0.0002
BMI 23–25	432/2183	1.00 (ref)	1.11 (0.67–1.84)	1.06 (0.56–2.00)	1.13 (0.43–2.97)	
BMI ≥ 25	1936/4432	1.00 (ref)	1.08 (0.82–1.42)	0.96 (0.67–1.38)	0.83 (0.48–1.45)	
Men						
BMI < 23	64/718	1.00 (ref)	1.71 (0.52–5.66)	4.10 (0.97–17.38)	6.89 (0.70–67.67)	0.4484
BMI 23–25	144/819	1.00 (ref)	0.53 (0.25–1.12)	0.27 (0.09–0.76)	0.20 (0.04–1.06)	
BMI ≥ 25	604/1778	1.00 (ref)	1.08 (0.72–1.63)	0.89 (0.50–1.57)	0.70 (0.28–1.70)	
Women						
BMI < 23	192/1550	1.00 (ref)	3.08 (1.20–7.93)*	3.11 (1.01–9.51)*	3.14 (0.65–15.23)	0.0023
BMI 23–25	288/1363	1.00 (ref)	1.76 (0.87–3.56)	2.44 (1.06–5.62)*	3.11 (0.90–10.68)	
BMI ≥ 25	1332/2654	1.00 (ref)	1.18 (0.82–1.69)	1.08 (0.68–1.72)	0.99 (0.48–2.01)	

*Note*: Adjusted for age, sex, education, smoking status, drinking status, and physical activity. Compared with reference group (nonexposed).

Abbreviations: BMI, body mass index.

* *p* < 0.05.

We further evaluated the joint effect of adulthood obesity and early life famine exposure on the development of MetS. The risk of MetS according to famine exposure status and obesity pattern defined by BMI is displayed in Table 4. Compared with the reference group (nonexposed participants with BMI < 23 kg/m^2^), the risk of MetS in overweight and obese participants increases accordingly in all famine‐exposed subgroups, using multivariable‐adjusted logistic regression analysis. Individuals who experienced famine exposure in the fetal stage and then developed general obesity during adulthood had a more than 17‐fold higher risk of MetS (OR 17.52; 95% CI, 10.07–30.48) compared to those who were neither obese nor famine exposed.

**TABLE 4:** Joint effect of adulthood obesity and early life famine exposure on risk of metabolic syndrome

	Case/total	Nonexposed	Famine exposure		
			Fetal	Childhood	Adolescence
Total					
BMI < 23	256/2268	1.00 (ref)	2.17 (1.13–4.17)*	2.72 (1.49–4.99)**	2.95 (1.45–6.02)**
BMI 23–25	432/2182	4.76 (2.60–8.72)**	5.21 (2.82–9.63)**	4.77 (2.63–8.65)**	4.87 (2.43–9.77)**
BMI ≥ 25	1936/4432	15.69 (9.10–27.02)**	17.52 (10.07–30.48)**	15.86 (8.86–28.36)**	14.10 (7.17–27.73)**
Men					
BMI < 23	64/718	1.00 (ref)	1.14 (0.39–3.30)	1.69 (0.68–4.17)	1.36 (0.44–4.19)
BMI 23–25	144/819	5.82 (2.50–13.51)**	3.68 (1.54–8.79)**	2.54 (1.06–6.10)*	2.51 (0.87–7.28)
BMI ≥ 25	604/1778	8.20 (3.86–17.45)**	9.00 (4.12–19.64)**	7.38 (3.18–17.14)**	5.92 (2.13–16.42)**
Women					
BMI < 23	192/1550	1.00 (ref)	3.32 (1.37–8.05)**	3.72 (1.60–8.67)**	4.20 (1.60–11.05)**
BMI 23–25	288/1363	3.98 (1.64–9.64)**	6.04 (2.52–14.49)**	6.77 (2.93–15.62)**	6.81 (2.63–17.63)**
BMI ≥ 25	1332/2654	20.36 (9.27–44.73)**	24.90 (11.25–55.10)**	23.99 (10.54–54.61)**	23.28 (9.17–59.06)**

*Note*: Adjusted for age, sex, education, smoking status, drinking status, and physical activity. Compared with reference group (nonexposed participants with BMI < 23 kg/m^2^).

Abbreviations: BMI, body mass index.

* *p* < 0.05.

** *p* < 0.01.

## 4 DISCUSSION

With 8883 Chinese adults from a community‐dwelling cohort who experienced the Great Chinese Famine during early life, we found that subjects exposed to famine during the fetal and childhood stages, especially women, tended to develop MetS in later life. In women, the coexistence of early life famine exposure and adulthood general obesity was associated with a higher risk of MetS in later life. Our research investigated for the first time the joint effect of early life famine exposure and adulthood general obesity on increasing the risk of MetS in later life.

Our current data were consistent with most previous studies on Chinese famines, demonstrating early life famine exposure increased the risk of MetS in later life. Studies based on Survey on Prevalence in East China for Metabolic Diseases and Risk Factors (SPECT‐China),^29^ the China Health and Nutrition Survey (CHNS),^30^ and the 2002 China National Nutrition and Health Survey (CNNHS)^12^ have reported that famine exposure in early life increased the risk of MetS in later life. Four other Chinese famine studies found similar results.^13^, ^14^, ^31^, ^32^ A meta‐analysis including 81 504 subjects indicated that, compared to the nonexposed group, individuals who experienced a famine during the fetal and childhood stages had an increased risk of MetS as adults.^33^ Our recent study further confirmed the strong relationship between early life famine exposure and the risk of developing MetS, as well as the gender difference, with a positive correlation in women but not in men. The fetal and childhood stages are periods when undernutrition is prone to have effects on people, which accelerates progression of metabolic disorder in adulthood. However, studies under the setting of famines in Europe, including the Siege of Leningrad Study and the Dutch Winter Famine Study, failed to demonstrate a positive association between famine exposure in early life and risk of MetS.^34^, ^35^ The rapid growth of the Chinese economy, the duration of the famine, and racial differences might be potential explanations for these inconsistent results.

In contrast to previous research, we found for the first time an interaction between famine exposure and BMI. Although the highest risk of MetS occurred in participants with both famine exposure and obesity, compared to the overweight and obese groups, the effect of famine exposure was more obvious in the lean group. Even if individuals maintain a healthy BMI in adulthood, famine exposure in early life does result in developing MetS. The results of our current study provide evidence supporting the essential famine exposure effect on metabolic disorders. Previous studies showed that the Chinese famine and general obesity in adulthood have no significant interaction on MetS risk.^13^, ^14^ Wang et al^14^ found that the interactions between the fetal‐, infant‐, and preschool‐exposed groups and BMI (grouped by 24 kg/m^2^) were not statistically significant in participants from CHARLS. Similarly, the Dongfeng‐Tongji cohort study^13^ did not observe any significant interaction between fetal and childhood famine exposure and BMI grouped by 24 kg/m^2^ on MetS risk. The differences among the current study and these two previous studies might be partly explained by the different population, sample size, and the definitions of MetS and BMI cutoff.

Since famine exposure during early life stages is known as a risk factor for MetS, it is worth clarifying whether the association between famine exposure and MetS risk differs between subjects with and without general obesity. By stratifying subjects according to BMI status, we examined the association between famine exposure and MetS risk within different BMI categories. We found that famine exposure did not have more harmful effects on MetS among participants with overweight and obesity than the lean population. The association between famine exposure during early life and the risk of MetS was observed only in the lean group but not in the overweight and obese groups. The inconsistent results among the three BMI categories could be partly caused by the distinct disturbance in the metabolic spectrum. For the lean group, famine exposure led to greater metabolic spectrum disturbance than for the overweight and obese groups. General overweight/obesity itself can increase the risk of metabolic disorder, and so this association may be masked to some extent. Furthermore, the strong effect of famine exposure on risk of MetS in the lean group may be partly explained by the fact that BMI can only represent general obesity rather than adipose tissue content and distribution, which could have effects on MetS risk.

The mechanisms of early life famine exposure and adulthood obesity on the development of MetS are still obscure. The mismatch between postnatal adequate nutrition and early life malnutrition might increase the risk of MetS in later life.^36^ First, as proven by a Dutch famine study, people with severe fetal undernutrition may be accompanied by reduced physical activity and increased fat intake in later life, resulting in increased total cholesterol and TG levels.^37^ The elevated blood lipid levels might increase the risk of MetS in the future. Second, animal models^38^ and human studies^39^ have provided evidence that undernutrition during the prenatal stage altered methylation levels of several genes associated with obesity and metabolic disturbance. In addition, animal studies found that early life undernutrition could result in an increased risk of the components of MetS in later life.^40^, ^41^, ^42^

What needs to be mentioned is the sex difference in the current study. Our results indicate that the increasing risk of MetS led by famine exposure could be aggravated by adulthood general obesity, which was more apparent in women compared with men. Such gender difference might be explained by several possible mechanisms. Females and males adapt differently during development. Sex steroids have a profound impact on the progression of metabolic diseases.^43^ A Dutch famine study observed an adverse prenatal environment caused permanent changes in the levels of DNA methylation, varied by gender.^44^ Furthermore, visceral adipose tissue presents a stronger association with the metabolic disorder in women than in men.^45^ Another possible mechanism to understand the underlying biological gender differences could be that visceral fat accumulation is particularly detrimental to women. Furthermore, as suggested by a previous study, the male‐sex preference culture in China may have masked the true health impact of famines on men^46^; thus, more significant long‐term effects of famines and other adversities on women than on men are often observed in such research. Moreover, the son preference culture may lead to a better health outcome for men.^46^ On the other hand, women may be more adaptable to famines than men. It has been shown that famine exposure was related to a lower sex ratio leading to more female babies.^47^ Thus, the increased survival of women may predispose them to MetS risk in adulthood.

The main strength of this study is the well‐characterized, representative cohort. Our cohort provides a sufficient number of MetS cases and limits sampling bias. Besides, the biochemical tests and detailed lifestyle factors we collected allowed us to adjust for potential confounding factors which can introduce substantial bias in the analysis. The anthropometric information was measured on site instead of being self‐reported, providing more accurate estimates of BMI. The Great Chinese Famine lasted much longer (about 3 years) and impacted a larger number of people compared with famines in other countries. Thus, we were able to take advantage of the data from this period to test our hypothesis.

This study has several limitations. First, the population of the current study was Chinese aged 40 years or above, so the conclusion may not have implications for younger and other ethnic populations. Second, some confounding factors were self‐reported. Information about eating habits and household income was also limited. In the period before China's reform, the Chinese government pursued an egalitarian policy, and the living standard was relatively low. Therefore, the limitation of socioeconomic factors might have a minor effect on the results of this study. Third, considering the nature of the current research as a cross‐sectional study, the causality between famine exposure during early life stages, obesity in adulthood, and the development of MetS cannot be elaborated. Fourth, the aging effect is an unavoidable issue in analyzing the association between health outcomes and famine exposure. The exposure groups in the famine cohort were classified according to the birth date of the participants, which cannot avoid the difference in age. The aging difference between individuals with and without famine exposure can introduce substantial bias in the analysis as the prevalence of MetS is highly correlated with aging. Fifth, given the limited geographic distribution of our study population, we could not analyze the severity of famine exposure and the risk of MetS. Besides, information about dietary patterns and parental weight status was lacking; thus, there may be biased results. Finally, the accurate dates of the beginning and the end of the Great Chinese Famine were unavailable; thus, misclassification of famine exposure subgroups might exist. However, the definition of famine exposure subgroups in the current study was aligned with the definition in former studies.^9^, ^10^, ^17^

In conclusion, we found that the coexistence of early life malnutrition and adulthood general overweight/obesity has an association with the increased risk of MetS in later life, especially in females. This finding may partially explain the emerging prevalence of MetS among Chinese adults who experienced chronic malnutrition in early life and suffer from obesity in later life. Our study supports preventive measures across the lifespan, from prenatal to late life, to mitigate the risk of MetS in the Chinese population.

## AUTHOR CONTRIBUTIONS

YZ, JL, and GN contributed to the study design and concept. YZ, HQ, and CH analyzed the data and drafted the manuscript. YZ, HQ, CH, SW, YZ, HL, LL, JZ, TW, ZZ, ML, YX, and MX contributed to data interpretation and the editing of the manuscript. YB, WW, YC, JL, and GN critically revised the manuscript for important intellectual content. All authors were involved in writing and revising the paper and had final approval of the submitted and published version. JL guarantees this work, has full access to the data, and takes responsibility for the integrity of the data.

## DISCLOSURE

The authors declare that the research was conducted in the absence of any commercial or financial relationships that could be construed as a potential conflict of interest.

## Supporting information


**Table S1**. ORs (95% CIs) for metabolic syndrome (defined by the modified Asian criteria) according to famine exposure in early life
**Table S2**. Components of the MetS according to famine exposure

**Figure S1**.
