# Supplementary material for: Association between early life famine exposure and risk of metabolic syndrome in later life
Source: J Diabetes. 2022 Sep 29;14(10):685–94. doi: 10.1111/1753-0407.13319 (PMC9574738; doi:10.1111/1753-0407.13319)
Supplement: Supplementary file 1 — Table S1. ORs (95% CIs) for metabolic syndrome (defined by the modified Asian criteria) according to famine exposure in early life Table S2. Components of the MetS according to famine exposure [file JDB-14-685-s002.docx]

**Supplementary Material**

**Supplementary Table 1. ORs (95% CIs) for metabolic syndrome (defined by the modified Asian criteria) according to famine exposure in early life**

|  |  | Famine exposure | | |
| --- | --- | --- | --- | --- |
|  | Nonexposed | Fetal | Childhood | Adolescence |
| **Whole cohort** |  |  |  |  |
| Case/Total | 328/1387 | 394/1225 | 1544/4029 | 949/2242 |
| Model 1 | 1.00 (ref) | **1.53 (1.29-1.82)** | **2.01 (1.75-2.31)** | **2.37 (2.04-2.75)** |
| Model 2 | 1.00 (ref) | 1.20 (0.98-1.46) | 1.11 (0.86-1.44) | 0.92 (0.61-1.38) |
| Model 3 | 1.00 (ref) | **1.24 (1.01-1.52)** | 1.12 (0.86-1.46) | 0.94 (0.63-1.42) |
| Model 4 | 1.00 (ref) | 1.18 (0.96-1.47) | 1.11 (0.83-1.48) | 0.94 (0.60-1.47) |
| **Men** |  |  |  |  |
| Case/Total | 133/540 | 121/464 | 346/1413 | 212/899 |
| Model 1 | 1.00 (ref) | 1.08 (0.81-1.44) | 0.99 (0.79-1.25) | 0.94 (0.74-1.21) |
| Model 2 | 1.00 (ref) | 0.99 (0.72-1.38) | 0.81 (0.52-1.28) | 0.67 (0.33-1.37) |
| Model 3 | 1.00 (ref) | 0.99 (0.71-1.39) | 0.81 (0.51-1.27) | 0.66 (0.32-1.34) |
| Model 4 | 1.00 (ref) | 0.98 (0.67-1.39) | 0.82 (0.51-1.31) | 0.68 (0.32-1.43) |
| **Women** |  |  |  |  |
| Case/Total | 195/847 | 273/761 | 1198/2616 | 737/1343 |
| Model 1 | 1.00 (ref) | **1.87 (1.50-2.33)** | **2.83 (2.37-3.38)** | **4.07 (3.35-4.93)** |
| Model 2 | 1.00 (ref) | **1.34 (1.05-1.72)** | 1.32 (0.95-1.82) | 1.11 (0.68-1.82) |
| Model 3 | 1.00 (ref) | **1.42 (1.10-1.83)** | 1.34 (0.97-1.86) | 1.16 (0.70-1.93) |
| Model 4 | 1.00 (ref) | **1.34 (1.01-1.79)** | 1.36 (0.94-1.97) | 1.17 (0.67-2.07) |

Note: Model 1: Unadjusted; Model 2: Adjusted for age and sex; Model 3: Adjusted for age, sex, education, smoking and drinking status and physical activity; Model 4: Further adjusted for body mass index.

**Supplementary Table 2.** **Components of the MetS according to famine exposure**

|  |  | Famine exposure | | |
| --- | --- | --- | --- | --- |
|  | Nonexposed | Fetal | Childhood | Adolescence |
| **Men** |  |  |  |  |
| **Central obesity** |  |  |  |  |
| Cases, n (%) | 16 (16.33) | 10 (10.20) | 44 (44.90) | 28 (28.57) |
| OR (95% CI) | 1.00 (ref) | 0.57 (0.18-1.78) | 0.70 (0.16-3.05) | 0.62 (0.06-5.94) |
| **High blood pressure** |  |  |  |  |
| Cases, n (%) | 350 (14.08) | 312 (12.56) | 1077 (43.34) | 746 (30.02) |
| OR (95% CI) | 1.00 (ref) | 0.71 (0.51-1.00) | 0.61 (0.38-0.97) | 0.46 (0.22-0.99) |
| **High triglycerides** |  |  |  |  |
| Cases, n (%) | 242 (19.79) | 187 (15.29) | 533 (43.58) | 261 (21.34) |
| OR (95% CI) | 1.00 (ref) | 0.73 (0.54-0.99) | 0.62 (0.41-0.95) | 0.38 (0.20-0.75) |
| **High fasting glucose** |  |  |  |  |
| Cases, n (%) | 141 (12.57) | 153 (13.64) | 478 (42.60) | 350 (31.19) |
| OR (95% CI) | 1.00 (ref) | 1.20 (0.87-1.64) | 1.07 (0.70-1.63) | 1.09 (0.56-2.10) |
| **Low HDL cholesterol** |  |  |  |  |
| Cases, n (%) | 171(19.39) | 152(17.23) | 354 (40.14) | 205 (23.24) |
| OR (95% CI) | 1.00 (ref) | 1.26 (0.91-1.75) | 1.16 (0.73-1.83) | 1.34 (0.65-2.77) |
|  |  |  |  |  |
| **Women** |  |  |  |  |
| **Central obesity** |  |  |  |  |
| Cases, n (%) | 97 (8.88) | 115 (10.53) | 534 (48.90) | 346 (31.68) |
| OR (95% CI) | 1.00 (ref) | 0.75 (0.48-1.19) | 0.94 (0.53-1.65) | 0.69 (0.19-1.62) |
| **High blood pressure** |  |  |  |  |
| Cases, n (%) | 391 (10.11) | 457 (11.81) | 1874 (48.44) | 1147 (29.65) |
| OR (95% CI) | 1.00 (ref) | 1.08 (0.84-1.40) | 0.86 (0.60-1.22) | 0.84 (0.47-1.50) |
| **High triglycerides** |  |  |  |  |
| Cases, n (%) | 154 (8.12) | 210 (11.07) | 980 (51.66) | 553 (29.15) |
| OR (95% CI) | 1.00 (ref) | **1.32 (1.00-1.75)** | **1.59 (****1.12-2.26)** | 1.29 (0.76-2.21) |
| **High fasting glucose** |  |  |  |  |
| Cases, n (%) | 118 (7.92) | 172 (11.55) | 699 (46.94) | 500 (33.58) |
| OR (95% CI) | 1.00 (ref) | **1.74 (1.29-2.35)** | **1.96 (****1.34-2.85)** | **2.91 (1.65-5.15)** |
| **Low HDL cholesterol** |  |  |  |  |
| Cases, n (%) | 353 (14.94) | 308 (13.04) | 1122 (47.50) | 579 (24.51) |
| OR (95% CI) | 1.00 (ref) | 0.78 (0.61-0.99) | 0.73 (0.53-1.00) | 0.61 (0.37-1.01) |

Multivariable model was adjusted for age, sex, education, smoking and drinking status, physical activity and BMI.
