# Supplementary figures and images for: Association between early life famine exposure and risk of metabolic syndrome in later life
Source: J Diabetes. 2022 Sep 29;14(10):685–94. doi: 10.1111/1753-0407.13319 (PMC9574738; doi:10.1111/1753-0407.13319)

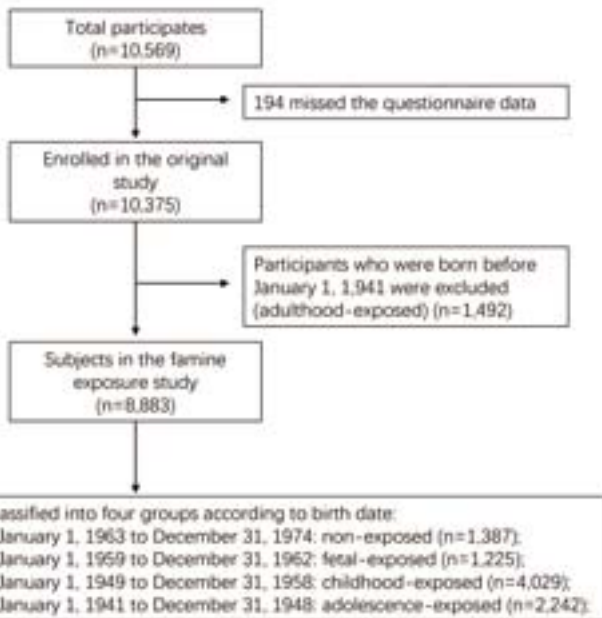

Supplement: Supplementary file 2 — Figure S1. [file JDB-14-685-s001.pdf]
